# Clinical significance of IgG antimitochondrial M2 antibody levels in primary biliary cholangitis: A single center study from China

**DOI:** 10.1371/journal.pone.0242164

**Published:** 2020-11-12

**Authors:** Lina Feng, Kaihui Dong, Xiaoxue Zhang, Bo Ma, Lin Chen, Qianqian Yang, Qingling Chen, Xiaoyu Wen, Qinglong Jin

**Affiliations:** 1 Department of Hepatology, The First Hospital of Jilin University, Changchun, China; 2 Department of Gastroenterology, Yantaishan Hospital, Shandong, China; Laval University, CANADA

## Abstract

**Background and objective:**

The relationship between antimitochondrial antibody (AMA) levels and the severity or prognosis of primary biliary cholangitis (PBC) is unclear. This study explored the clinical significance of serum IgG antimitochondrial M2 antibody (IgG-M2) levels.

**Methods:**

From 2008 to 2017, a retrospective analysis was conducted with PBC patients who had available quantitative values of serum IgG-M2 levels obtained with ELISA based on triple expression hybrid clones. The patients were divided into two groups based on high and low concentrations of IgG-M2. Baseline parameters, the incidence of adverse events, and prognosis were compared.

**Results:**

Among the 530 PBC patients, the levels of albumin, cholinesterase, hemoglobin, fibrinogen and triglycerides and the red blood cell count were significantly lower in the high-concentration group than in the low-concentration group (n = 263, 49.6%). The red cell distribution width (RDW) and levels of serum immunoglobulin (Ig) G, IgM and IgA were significantly higher in the high-concentration group than in the low-concentration group. Spearman’s correlation analysis suggested that the correlation between the above baseline indicators and IgG-M2 levels was statistically significant but weak (r < 0.2, P < 0.05). In total, 203 patients were followed up, of whom 87 (42.9%) were in the high-concentration group. During the median follow-up period of 52 months (range: 28–75), 121 (59.6%) experienced hepatic decompensation, and 37 (18.2%) died or underwent liver transplantation. There was no significant difference in the incidence of complications or survival (log-rank test: P = 0.079) between the two groups. One year after ursodeoxycholic acid (UDCA) treatment, the two groups had similar responses. In addition, the levels of IgG-M2 did not fluctuate significantly during treatment.

**Conclusion:**

IgG-M2 levels were not related to the disease severity, prognosis or efficacy of UDCA. The levels of IgG-M2 did not change significantly during treatment.

## Introduction

Primary biliary cholangitis (PBC) is a progressive cholestatic liver disease that eventually develops into liver fibrosis and cirrhosis [1]. It is common in middle-aged and old women, and there is no definite treatment except liver transplantation in the advanced stage. The etiology of PBC is still largely unclear. At present, most researchers believe that it is caused by the interruption of mitochondrial antigen tolerance due to the exposure of genetically susceptible individuals to specific environmental factors, leading to the autoimmune-driven selective destruction of small and medium bile duct epithelial cells in the liver and cholestasis [2, 3].

Antimitochondrial antibodies (AMAs) are the characteristic hallmarks of PBC and are divided into 9 subtypes. Among them, the M2 subtype is the most specific for the diagnosis of PBC. The target autoantigens in PBC patient sera have been identified as the E2 subunits of the pyruvate dehydrogenase complex (PDC-E2), the branched-chain 2-oxo-acid dehydrogenase complex (BCOADC-E2), and the 2-oxoglutarate dehydrogenase complex (OGDC-E2) [4, 5]. Although PDC-E2 is the major autoantigen in PBC, approximately 10% of PBC patients only react to BCOADC-E2 and/or OGDC-E2 [6]. Compared with IFA or conventional PDC-E2-based ELISA, detection based on the triple expression hybrid clone (MIT3) containing the immunodominant epitopes of these three antigens had superior performance [7].

In the past few decades, although the relationships between AMA levels and the severity, liver histology, biochemistry and prognosis of PBC have been widely studied, the results are still controversial. In addition, most of the studies were based on traditional detection techniques, and quantitative results were not available. Especially in mainland China, there has been almost no research performed on the clinical significance of M2 levels. In the present study, we discuss the significance of IgG-M2 levels from three perspectives: the baseline index, prognosis and treatment.

## Methods

### Study population

This study was approved by the Ethics Committee of the First Hospital of Jilin University. All participants provided written informed consent. All data were completely anonymized before analysis. We retrospectively examined the information from patients diagnosed with PBC at our institution between January 2008 and November 2017. PBC was diagnosed when at least two of the three following criteria were fulfilled: 1) biochemical evidence of cholestasis, for instance, elevated alkaline phosphatase (ALP) levels; 2) positivity for M2 (>20 U/ml); and 3) compatible liver histology [8]. Patients were excluded if 1) they had viral hepatitis, alcoholic liver disease or other autoimmune liver diseases; 2) they had concomitant malignant tumors in other organs; or 3) they had unavailable or insufficient clinical data.

### Baseline characteristics and follow‐up procedures

**Fig 1:**
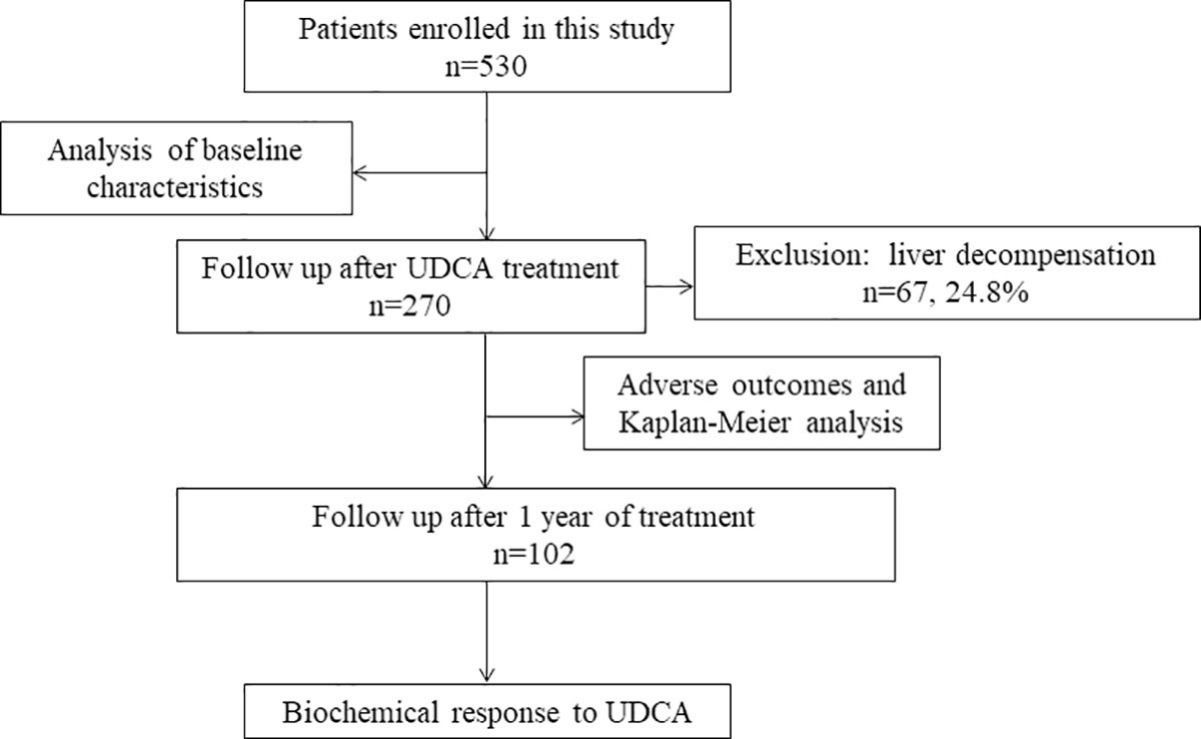
Patient flow chart.

A total of 530 patients with PBC were analyzed retrospectively, and standard techniques were used to evaluate the liver biochemical indexes, hematological indexes, liver pathology, clinical stages, and levels of serum lipids, IgA, IgG and IgM. The serum concentration of IgG-M2 was determined with the enhanced performance M2 ELISA [M2 EP (MIT3) ELISA, QUANTA Lite, Inova Diagnostics, and San Diego, CA]. After excluding patients with liver decompensation (n = 67), 203 patients who were treated with ursodeoxycholic acid (UDCA) for more than 6 months and had available follow-up information in the inpatient medical records were further analyzed with regard to the occurrence of adverse events, such as variceal bleeding, ascites, hepatic encephalopathy (HE), hepatocellular carcinoma and spontaneous bacterial peritonitis (SBP). The start date was defined as the date of the diagnosis of PBC, and the follow-up deadline was April 2020. A total of 102 patients had follow-up data throughout 1 year of treatment (Fig 1). The diagnosis of cirrhosis was mainly based on imaging. Decompensated cirrhosis was defined as the occurrence of liver-related complications, such as ascites, variceal bleeding, or hepatic encephalopathy. Histological staging was performed based on the standard published by Ludwing et al. [9].

### Statistical analysis

The Kolmogorov-Smirnov test was applied to test the normality of the distribution of variables. Variables with normal distributions are represented by the means ± standard deviation (SD), while those without a normal distribution are reported as the medians (interquartile ranges (IQRs)). T-tests were used for the comparison of normally distributed continuous variables, and the Mann–Whitney U-test was used for nonnormally distributed variables. Spearman correlation analysis was used to investigate the correlations between IgG-M2 levels and the observational data. IgG-M2 levels before and after treatment were compared with the Wilcoxon matched-pairs rank-sum test.

Categorical variables are represented as counts and percentages, and the chi-squared test was used for comparisons. Transplant-free survival (TRS) was assessed using the Kaplan–Meier method and compared using log-rank tests. A two-sided P value < 0.05 was considered significant. Data analyses were performed with SPSS statistics version 23.

## Results

### Demographics and baseline examinations (530 patients)

The included 530 patients had a mean age of 58.7 years at baseline. The male-to-female ratio was 1:5.6. Eighteen patients (3.4%) were negative for IgG-M2 (< 20 U/ml). According to the median serum IgG-M2 concentration (185.0 U/ml), the patients were divided into two groups: 267 patients (50.4%) were included in the high-concentration group (≥185 U/ml), and 263 patients (49.6%) were included in the low-concentration group (<185 U/ml). As shown in Table 1, the levels of albumin, cholinesterase, hemoglobin, fibrinogen and triglyceride and the red blood cell (RBC) count were lower in the IgG-M2 high-concentration group than in the low-concentration group. The red cell distribution width (RDW) and the levels of IgA, IgG and IgM were higher in the high-concentration group than in the low-concentration group (P < 0.05). Liver pathology results were available in 60 patients. No significant difference was found between the two groups (*P* = 0.739). Spearman’s correlation analysis suggested that the levels of albumin, cholinesterase, hemoglobin, fibrinogen and triglyceride and the RBC count were negatively correlated with the IgG-M2 level (r = -0.139, -0.174, -0.116, -0.115, -0.123, and -0.168, respectively, P<0.05), and the RDW and the levels of IgG, IgA and IgM were positively correlated with the IgG-M2 level (r = 0.110, 0.217, 0.116, and 0.196, respectively, P<0.05) (Table 2).

**Table 1:** Demographics and baseline characteristics of 530 patients with PBC.

	Total (n = 530)	IgG-M2<185 U/ml (n = 263)	IgG-M2≥185 U/ml(n = 267)	P-value
Age, years	58.7±11.9	58.1±12.8	59.2±10.8	0.308
Female, n (%)	450(84.9)	222(84.4)	228(85.4)	0.752
AST, U/L	53.1(34.6–89.0)	52.8(34.8–89.0)	53.6(34.0–91.0)	0.913
ALT, U/L	42.0(24.3–85.1)	43.3(24.3–99.5)	39.8(24.2–77.5)	0.143
ALP, U/L	178.8(111.9–328.9)	177.0(116.0–302.0)	182.0(104.0–357.4)	0.486
γ-GT, U/L	156.5(65.23–373.8)	159.8(75.4–356.0)	142.0(61.5–388.0)	0.363
ALB, g/L	32.3±6.2	33.1±6.0	31.7±6.3	0.009
GLO, g/L	32.8(29.0–37.2)	32.0(28.3–36.1)	33.3(30.0–38.4)	0.010
A/G	1.0±0.3	1.2±0.3	1.0±0.3	0.001
TBIL, μmol/L	23.8(13.4–58.3)	22.1(12.3–56.4)	26.4(13.8–60.7)	0.112
ChE, U/L	4539.0(2776.8–6493.8)	4969.0(3059.0–7015.0)	4010.0(2630.0–6181.0)	0.001
WBC count, 10 ^9^/L	4.8(3.5–6.5)	4.7(3.3–6.2)	4.8 (3.5–7.0)	0.076
RBC count, 10 ^12^/L	3.7(3.1–4.2)	3.8(3.2–4.3)	3.5(3.0–4.1)	0.000
Hemoglobin, g/L	111.5(91.0–125.0)	114.0(94.0–127.0)	108.0(88.0–124.0)	0.020
RDW, %	15.0(13.7–16.6)	14.6(13.5–16.4)	15.2(13.8–16.6)	0.048
PLT, 10^9^/L	137.0(80.0–209.0)	147.00(85.0–208.0)	126.0(74.0–214.0)	0.171
RDW/Plt	0.1(0.1–0.2)	0.1(0.1–0.2)	0.1(0.1–0.2)	0.111
FBG, g/L ^a^	2.5(1.9–3.0)	2.5(2.0–3.1)	2.4(1.7–3.0)	0.047
APTT, s^a^	34.6(30.7–38.2)	33.8(30.2–37.4)	35.3(31.2–39.1)	0.007
PT, s^a^	11.8(10.6–13.2)	11.7(10.8–13.3)	11.8(10.5–13.1)	0.896
PTA, %^a^	91.3±24.4	92.8±23.4	90.0±25.2	0.197
Triglycerides, mmol/L^b^	1.1(0.8–1.7)	1.2(0.8–1.8)	1.0(0.7–1.5)	0.008
Total cholesterol, mmol/L^b^	4.4(3.2–5.7)	4.4(3.4–5.8)	4.3(3.1–5.6)	0.256
IgM, g/L ^c^	2.8(1.8–4.8)	2.5(1.6–4.2)	3.1(2.0–5.3)	0.004
IgA, g/L^c^	3.2(2.4–4.6)	3.0(2.3–4.3)	3.5(2.5–5.0)	0.024
IgG, g/L^c^	15.7(12.9–19.5)	14.5(12.3–18.1)	16.6(13.7–21.0)	0.000
Cirrhosis, n (%)	292(55.1)	139(52.9)	153(57.3)	0.303
Histological stage, n (%)				0.739
I–II	30(50.0)	24(49.0)	6(54.5)	
III–IV	30(50.0)	25(51.0)	5(45.5)	

P values refer to comparisons between the high- and low-concentration groups. ALB, albumin; ALP, alkaline phosphatase; ALT, alanine aminotransferase; APTT, activated partial thromboplastin time; AST, aspartate aminotransferase; A/G, albumin/globulin; ChE, cholinesterase; FBG, fibrinogen; GLO, globulin; IgA, immunoglobulin A; IgG, immunoglobulin G; IgM, immunoglobulin M; IgG-M2, IgG antimitochondrial M2 antibody; PLT platelet; PT, prothrombin time; PTA, prothrombin activity; RBC, red blood cell; RDW, red cell distribution width; TBIL, total bilirubin; WBC, white blood cell; γ-GT, gamma-glutamyl transpeptidase.

^a^ total of 514 patients had FBG, APTT, PT and PTA data.

^b^ A total of 413 patients had triglyceride and total cholesterol data.

^c^ A total of 359 patients had IgM, IgA and IgG data.

**Table 2:** Spearman’s correlation analysis between baseline indicators and IgG-M2 levels.

	r-value	P-value
ALB, g/L	-0.139	0.001
ChE, U/L	-0.174	<0.001
RBC count, 10 ^12^/L	-0.168	<0.001
Hemoglobin, g/L	-0.116	0.008
RDW, %	0.110	0.011
Triglycerides, mmol/L	-0.123	0.012
FBG, g/L	-0.115	0.009
IgA, g/L	0.116	0.028
IgG, g/L	0.217	<0.001
IgM, g/L	0.196	<0.001

r values refer to the Spearman’s correlation coefficient. ALB, albumin; ChE, cholinesterase; FBG, fibrinogen; PLT platelet; RBC, red blood cell; RDW, red cell distribution width; IgA, immunoglobulin A; IgG, immunoglobulin G; IgM, immunoglobulin M.

### Adverse outcomes and survival of UDCA-treated primary biliary cholangitis patients (203 patients)

Patients with liver decompensation (n = 67, 24.8%) were excluded, and the follow-up data from 203 patients who received UDCA for more than 6 months were analyzed. The median follow-up period was 52 months (interquartile range: 28–75 months). Ninety-seven (47.8%) patients had liver cirrhosis at the time of diagnosis. During follow-up, 121 patients (59.6%) had liver-related adverse outcomes. Ascites (41.9%) was the most common complication, followed by variceal bleeding (11.8%), HE (3.9%), liver cancer (n = 3, 1.5%) and SBP (n = 1). There was no significant difference in the incidence of complications between the two groups (Table 3). Thirty-seven patients (18.2%) experienced terminal events. Among them, 6 patients underwent liver transplantation, and 31 died of liver-related causes (variceal bleeding n = 14, HE n = 9, SBP or severe infection n = 4, liver failure n = 3, rupture and hemorrhage of liver cancer n = 1).

**Table 3:** Comparison of the incidence of liver-related adverse events in PBC patients with different IgG-M2 levels.

	Outcomes (n = 203)	IgG-M2<185 U/ml (n = 116)	IgG-M2≥185 U/ml (n = 87)	P-value
Cirrhosis (at diagnosis)	97(47.8)	49(42.6)	48(54.5)	0.068
Ascites	85(41.9)	42(36.5)	43(48.9)	0.077
Variceal bleeding	24(11.8)	11(8.2)	13(14.4)	0.255
Encephalopathy	8(3.9)	6(5.9)	2(2.5)	0.285

All data are presented as n (%).

**Fig 2:**
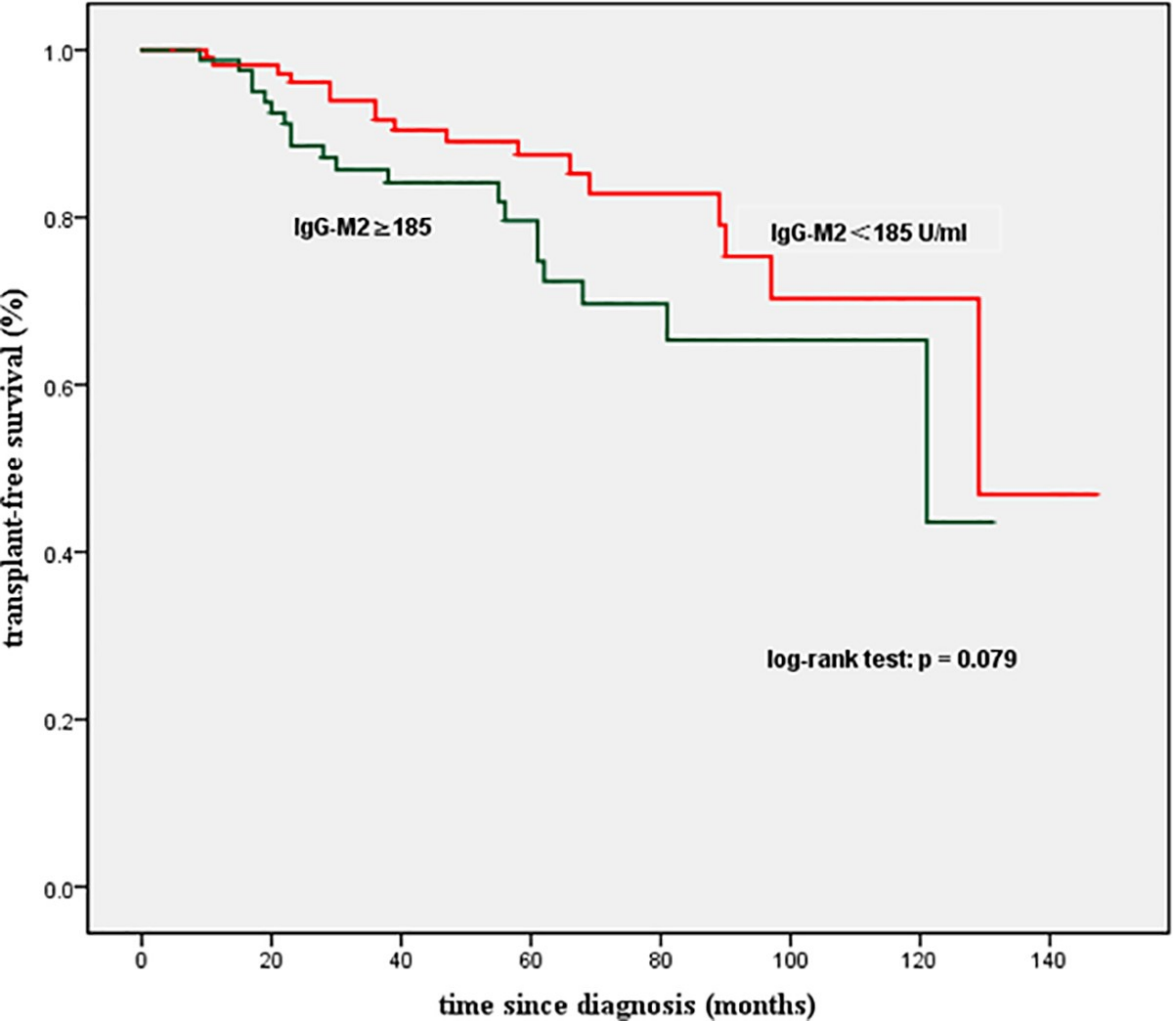
Kaplan–Meier plots for transplant-free survival among patients with PBC stratified by IgG-M2 levels. No significant difference was observed (P<0.05). IgG-M2, IgG antimitochondrial M2 antibody.

Fig 2 shows the long-term prognoses in the two groups, with calculated mean survival times of 118.0 months and 99.2 months, respectively. However, there was no significant difference in TRS between these two groups (p-value based on log-rank test = 0.079).

### Biochemical response to UDCA (102 patients)

In total, 102 UDCA-treated patients had follow-up information after 1 year of treatment. The response to UDCA was evaluated based on the Paris I criteria. There was no significant difference between the high- and low-concentration groups (p = 0.501) (Table 4).

**Table 4:** Comparison of response to UDCA in PBC patients with different IgG-M2 levels.

	IgG-M2<185 U/ml (n = 62)	IgG-M2≥185 U/ml(n = 40)	P-value
Intolerance	33(53.2)	24(60.0)	0.501
Response	29(46.8)	16(40.0)	

All data are expressed as n (%).

### Trend in IgG-M2 levels over time

**Fig 3:**
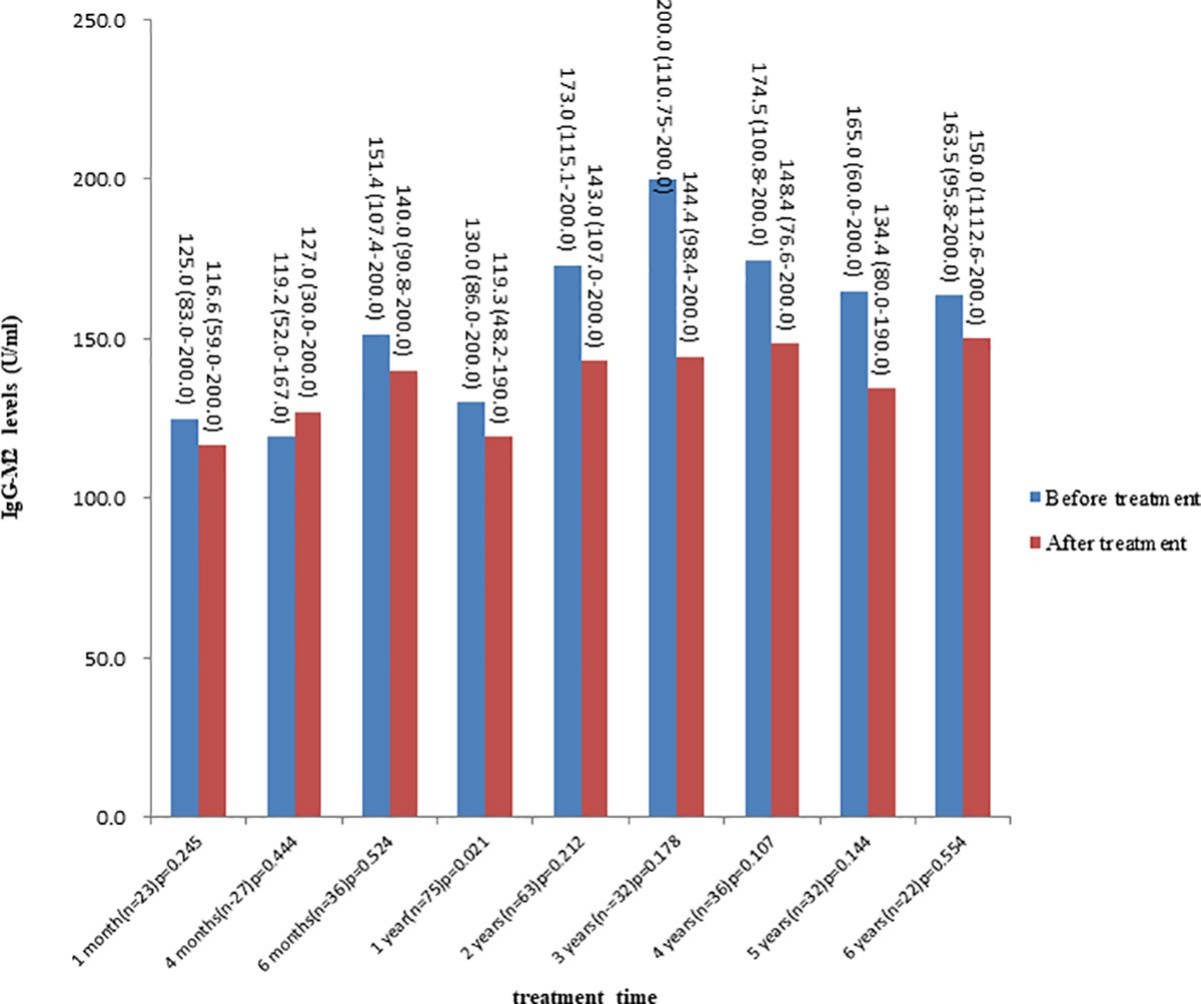
Comparison of IgG-M2 levels before and after UDCA treatment. All data are reported as the median (IQR).

Fig 3 showed the comparisons of the IgG-M2 values after 1 month, 4 months, 6 months, 1 year, 2 years, 3 years, 4 years, 5 years and 6 years of UDCA treatment with the respective baseline values. (values> 200 U/ml were reported as 200 U/ml, values < 20 U/ml were reported as 20 U/ml). No significant differences were found in serum IgG-M2 concentration before and after treatment, except that the IgG-M2 levels decreased after one year (median, 130.0 vs 119.3; IQR, 86.0–200.0 vs 48.2–190.0; P = 0.021).

## Discussion

To date, whether the levels of AMAs are related to the severity and prognosis of PBC has been controversial. As early as 1980, Christense et al. studied 236 patients with PBC and found that AMA titers increased with the progression of the disease [10]. In 1983, Roll et al. observed that PBC patients with higher AMA titers had more severe symptoms [11]. In addition, some studies suggested that a high titer of AMAs was related to the histological progress of PBC [11, 12]. However, Van Norstrand et al. found that the level of AMAs could not be used to predict the progression of PBC [13]. Similarly, most studies have shown that AMA status and AMA profiles are not prognostic factors [14]. With the emergence of quantitative tests, Flisiak et al. showed that the M2 concentration was significantly correlated with the Mayo score and bilirubin and albumin levels in 2005 [15]. In 2007, Gabeta et al. used the same method to detect IgG-AMAS in PBC patients. IgG-AMA-positive patients had more severe histological manifestations and higher levels of ALP, IgG and IgM. The AMA titer was correlated with the levels of ALP, IgG, IgM, and albumin (r = +0.219, r = +0.332, r = +0.430, r = -0.221, respectively, P<0.05) [7]. Alfano et al. grouped 90 PBC patients according to EliA-M2-IgG levels and pointed out the feasibility of using quantitative tests to monitor disease activity and perform risk stratification [16]. In our study, the levels of ALB, cholinesterase, triglycerides and fibrinogen in the high-concentration group were significantly lower than those in the low-concentration group, but there were no significant differences in liver enzyme levels, histology or clinical stage. We hypothesized that the low levels of these substances may be related to the higher incidence rate of cirrhosis and the decline in liver synthetic functions in the high concentration group. Spearman correlation analysis was further used to compare the quantitative detection results for IgG-M2 and the baseline indicators. The data showed that the correlation between them was statistically significant but weak (r < 0.2, P < 0.05), which was similar to the results obtained in the study by Gabeta.

In addition, many researchers have compared the immunoglobulin levels between AMA-positive and -negative patients with PBC. Most results showed that the serum IgM level in AMA-negative patients was lower than that in AMA-positive patients [17, 18]. In our results, the levels of immunoglobulins (including IgG, IgA, IgM) in the high-concentration group were significantly higher than those in the low-concentration group, suggesting that there may be differences in immune status between AMA-positive and AMA-negative PBC patients and between patients with high and low levels.

Among the 203 patients without complications who completed follow-up, 47.8% had cirrhosis at baseline, and the median follow-up time was 52 months (interquartile range: 28–75 months). The 5-year and 10-year TRS rates were 84.1% and 67.6%, respectively, which were consistent with the results of two other studies in China [19, 20] but lower than those reported by the UK-PBC group [21]. By the end of follow-up, 121 patients (59.6%) had developed liver decompensation. Ascites, variceal bleeding and HE were common complications of PBC. The reasons for the higher complication rates and lower survival rates in our cohort may be as follows: 1) the current cohort included a significant proportion of patients with advanced liver disease. The proportion of patients with compensated and decompensated cirrhosis was higher than that in a study conducted in Beijing [19] and that in a large cohort study conducted in Europe [22]. This may be because people in mainland China, especially those in less developed areas such as the northeast, do not have a high level of awareness of this disease. Many patients do not visit a doctor until they have advanced symptoms, unlike in Western countries, in which approximately 50–60% of patients are asymptomatic at the time of diagnosis [23]. 2) The patients who completed follow-up were mainly inpatients, and most of them had more severe symptoms and signs than outpatients. 3) There are ethnic and regional differences in the clinical manifestations, progression and prognosis of PBC [20, 24].

Moreover, there was no significant difference in the incidence of liver-related complications between the high- and low-concentration groups. The survival times of the two groups were similar (P = 0.079 according to the log-rank test), whereas Gunnar et al. found that the rate of survival free from liver-related complications (including death and transplantation) in AMA-negative patients was significantly lower than that in AMA-positive patients [25]. However, most previous reports indicated no differences in the prognosis of PBC patients who were positive and negative for AMAs [26].

UDCA is still recognized as the first-line treatment for PBC, although up to 40% of patients have a poor response. Most previous studies suggested that the AMA status would not affect the response of PBC patients to UDCA [27, 28]. The results of this study showed that the levels of IgG-M2 did not affect the therapeutic response. Furthermore, IgG-M2 levels did not change significantly during UDCA treatment, which was in line with the results of Invernizzi [29] and Benson [30]. Therefore, there is no need to monitor IgG-M2 levels during the course of treatment, as it would impose an unnecessary economic burden on patients.

In the past, most studies on AMAs used traditional methods, such as IFA, and mainly focused on the differences between AMA-negative and AMA-positive patients with PBC. Except for their diagnostic value, the significance of AMAs is still uncertain. With the emergence of new detection methods, we were able to adopt a more accurate quantitative method to determine the IgG-M2 level in PBC patients in our hospital. In conclusion, this cohort study showed that IgG-M2 levels were not associated with disease severity, disease prognosis or the efficacy of UDCA. IgG-M2 levels did not change significantly during treatment. In addition, nearly half of the patients in this study suffered from liver cirrhosis at the time of diagnosis, while asymptomatic patients were clinically rare. With continued social and economic development, we think this situation may improve in the future.

Our study has several limitations. First, as this was a single-center retrospective study, all of our analyses were limited to the recorded data. Second, the M2 value was limited by the detection instrument, and the upper limit could only be expressed as greater than 200 U/ml. Third, as one of the largest tertiary medical centers for infectious and liver diseases in Northeast China, our data may not be representative of the general population due to referral bias and geographic constraints. Nevertheless, the study systematically described the significance of serum IgG-M2 levels in PBC patients. We hope that this information can serve as a useful reference for clinicians.

## Supporting information

S1 Data 530 patients. (SAV)

S2 Data 171 patients. (SAV)

S3 Data 203 patients. (SAV)

S4 Data Trend of IgG-M2 levels over time. (SAV)

S1 File STROBE statement—checklist of items that should be included in reports of observational studies. (DOCX)
